# Modulation of the Hoffmann reflex in the tibialis anterior with a change in posture‡

**DOI:** 10.14814/phy2.14179

**Published:** 2019-07-17

**Authors:** Janelle Unger, Justin W. Andrushko, Alison R. Oates, Doug W. Renshaw, Trevor S. Barss, E. Paul Zehr, Jonathan P. Farthing

**Affiliations:** ^1^ College of Kinesiology University of Saskatchewan Saskatoon Saskatchewan Canada; ^2^ Rehabilitation Neuroscience Laboratory, Physical and Health Education University of Victoria Victoria British Columbia Canada

**Keywords:** H‐reflex, postural control, reflex modulation

## Abstract

Hoffmann (H‐) reflex amplitudes in plantar flexor soleus muscle are modulated by posture, yet dorsiflexor tibialis anterior (TA) H‐reflex parameters have sparingly been studied. The purpose was to investigate modulation of the TA H‐reflex when postural demands are increased from sitting to standing. In this study, data from 18 participants (Age: 25 ± 4 years, Height: 170.9 ± 9.5 cm, Weight: 75.9 ± 17.2 kg) allowed comparison of two experimental conditions involving different postures (i.e. sitting and standing). Maximal amplitude of the TA H‐reflex (*H*_max_) as a percent of the maximal M‐wave amplitude (*M*_max_) (*H*_max_ (% *M*_max_)) during sitting and standing was compared using ANOVA. Modulation of TA H‐reflex amplitude was found: Eleven participants showed facilitation and seven showed no change of reflex amplitudes. Only participants in the facilitation group showed modulation related to changes in posture (sitting: 8.7 ± 2.9%; standing: 14.8 ± 6.7%, *P* = 0.005). These data provide evidence of the sensitivity to posture of TA H‐reflexes. As with task‐dependent changes in soleus H‐reflexes, presynaptic regulation of Ia afferent transmission is a possible mechanism. Further investigations into causes of modulation are warranted.

## Introduction

Hoffmann (H‐) reflex amplitude modulation has been used to study the relationship between Ia afferent feedback and postural control (Chen and Zhou, 2011). Presynaptic inhibition is an important mechanism modulating the H‐reflex amplitude, and it changes with alterations in posture (McNeil et al., 2013). Presynaptic inhibition is positively correlated with increases in postural demand, such as lying to standing, and standing to walking (Zehr, 2002). Changes in presynaptic inhibition as inferred from modulation of H‐reflex amplitude when background electromyography (EMG) and other factors are controlled, suggest a shift in the central nervous system (CNS) to increase voluntary control over the body and reduce fall risk (Huang et al., 2009). However, changes can be seen in presynaptic inhibition without correlating H‐reflex modulation when the H‐reflex is conditioned in different ways, meaning that presynaptic inhibition is not the only mechanism modulating the H‐reflex (Johannsson et al., 2015; Pavailler et al., 2016). Postactivation depression disynaptic inhibitory pathways, such as Ib inhibitory interneurons and Renshaw cells, allow inhibitory postsynaptic potentials to suppress motorneurons (Pierrot‐Deseilligny and Mazevet, 2000).

The ankle plantar flexor muscle, soleus (SOL), is the most commonly targeted muscle for H‐reflex studies (Zehr, 2002). Prior research shows SOL H‐reflex amplitudes decrease when postural demand increases; attributed to maintaining postural stability while standing (Chen and Zhou, 2011; Kim et al., 2012, 2013). In contrast, there have been limited investigations into H‐reflex modulation of the tibialis anterior (TA) muscle (Dragert and Zehr, 2009; Tallent et al., 2013). This is surprising since the TA is a key muscle for maintaining balance due to its role in proprioception (Di Giulio et al., 2009) and ankle control during posture (Obata et al., 2014) and locomotion (Hamner et al., 2010). Understanding TA H‐reflex modulation could have functional applications for clinical work in assessing spinal and supraspinal contributions to stability and locomotion in individuals with neurological impairment such as stroke, spinal cord injury, Parkinson’s disease, or multiple sclerosis. However, TA H‐reflex amplitudes have been shown previously to exhibit bidirectional modulation (Dragert and Zehr, 2009), possibly due to differences in Ia presynaptic inhibition “set point” or basal levels. Further research involving posture changes and TA H‐reflexes is needed.

As a first step in understanding TA H‐reflexes and changes in posture, the purpose of this experiment was to examine modulation of TA H‐reflex amplitudes between two very simple posture conditions (i.e. sitting and standing) in healthy, young adults. We hypothesized that the H‐reflex amplitude would be suppressed in the TA when comparing standing to sitting, following the same pattern as previously shown in the SOL H‐reflex (Chen and Zhou, 2011; Kim et al., 2012, 2013).

## Materials and Methods

### Ethical approval

This study was approved by the University of Saskatchewan Research Ethics Board (Biomedical protocol # 16‐16) in accordance with the Declaration of Helsinki (1964) except for registration in a database. All participants provided informed written consent.

### Participants

Twenty‐nine participants were recruited, and a final sample of 18 participants were included in the full analysis (male *n* = 10, female *n* = 8, 24 ± 4 years, weight: 75.9 ± 17.2 kg height: 170.9 ± 9.5 cm). One participant stopped due to onset of nausea from the electrical stimulation and 10 others were excluded from analysis due to a lack of a detectable H‐reflex and/or M‐wave contamination from the stimulation artifact. We expected to not elicit H‐reflexes in all participants, as it has been previously reported that H‐reflexes can be difficult to elicit in the TA, with up to 11% of success (Zehr, 2002); however, our proportion (18/29 or 62%) was significantly higher than this value. Individuals were excluded if they reported previous injuries to peripheral nerves that could affect transmission of afferent information (Laurin et al., 2009), any neurological conditions; current pain or injury in the right knee, ankle, or lower leg; or history of a grade two sprain (partial tear of the ligament) or higher in the right knee or ankle.

### Protocol

Electromyography (EMG) electrodes (Delsys Inc., MA) were placed on the TA and the SOL muscles of the right leg, consistent with published recommendations (Zehr, 2002; Misiaszek, 2003; Kim et al., 2013). Background EMG activity was found to be consistent between the TA and SOL muscles (see results). Custom software in Labview (Version 8.6 National Instruments, US) was used to obtain stimulator pulses and EMG data. All channels were acquired at a sampling rate of 1000 Hz. Analog signals from each device were converted to digital signals displayed in Labview on a desktop computer and recorded for analysis. Participants performed three maximum voluntary isometric contractions (MVC) of the right TA (dorsiflexion) against the resistance of the researcher (Wilson and Murphy, 1996). While seated with 90 degrees of knee flexion, and the foot flat on the floor, participants were instructed to dorsiflex their right foot against the researcher’s resistance, provided manually, as hard as possible for 3 sec. Verbal encouragement was provided, and 1‐minute rest was given between each MVC. The peak EMG amplitude from the MVC tests was used to set a background contraction where appropriate (explained below).

After MVC testing, the optimal stimulation location was determined by placing bipolar electrodes on the surface of the leg over the deep fibular nerve, distal to the anticipated bifurcation of the common fibular nerve near the fibular head. A high voltage constant current electrical stimulator (DS7AH, Digitimer, Ltd., Bedfordshire, UK) delivered single pulses (0.5 msec) with stimulation intensities ranging from 15 to 20 milliamps (mA) to find the optimal location. The electrodes were considered to be in the correct location when the stimulation evoked caused visible contraction of the TA that caused dorsiflexion of the foot. This location was confirmed during standing as the skin may move relative to the nerve with the change in posture.

After the stimulation location was determined, each participant was tested under two postural conditions (i.e. sitting or standing) with and without a mild background contraction of the TA (i.e. EMG levels quiet or 10% MVC). The background contraction condition was included as it has been previously reported that H‐reflexes can be difficult to elicit at rest (Pierrot‐Deseilligny and Mazevet, 2000; Zehr, 2002), and 10% was chosen as the level based on previous literature (Dragert and Zehr, 2009). Since we were unable to detect H‐reflexes at rest, analyses were only performed on the sitting and standing conditions with the background contraction. The four conditions were administered in semi‐random order, alternating between quiet trials, and trials with a background contraction, to avoid fatigue. During the sitting trials, the ankle angle was matched to the angle during standing (Frigon et al., 2007). In both standing and sitting, the *M*_max_ was determined by stimulating at higher intensities (10–50 mA) until a plateau or slight decrease in the *M*_max_ amplitude was observed (Zehr, 2002). A supramaximal stimulation intensity (current [mA] at *M*_max_ + 10%) was used as the maximum stimulation intensity tested during data collection to ensure a maximum M‐wave was recorded. An H‐reflex and M‐wave recruitment curve were built for each experimental condition, beginning with a minimum level of current (range 1–5 mA) and progressing up by 1mA increments until the maximum M‐wave response (up to 50 mA) was elicited. One trial consisted of five stimulations at each intensity, delivered at random intervals within a 3–5 sec window controlled by the Labview software. Stimulations occurred at least 3 sec apart to avoid postactivation depression (Zehr, 2002). EMG activity was recorded in both the TA and SOL, and the average of the five recordings at each intensity was used to reduce variability (Zehr, 2002; Chen and Zhou, 2011; Kim et al., 2012, 2013; McNeil et al., 2013). For the trials involving a background contraction, a live visual representation (bar graph) of the EMG signal on a scale of 0‐100% (where 100% represented their MVC) was used as biofeedback. Participants were instructed to hold a 10% MVC contraction as indicated by matching their EMG activity to a target line on the bar graph displayed in front of them on a computer monitor.

Finally, muscle activity prior to stimulation was determined by taking the mean absolute value of the EMG signal from the start of the recording up to the stimulation artifact and used as a proxy of similar synaptic drive to the α‐MN between antagonistic pairs.

### Data analyses

Analyses were only performed on the sitting and standing conditions with background contractions, as we were unable to detect H‐reflexes at rest. For each stimulation intensity, the averages of the five stimulations were filtered using a dual‐pass, low‐pass Butterworth filter with a cutoff at 100 Hz. If an M‐ or H‐reflex was present, the peak‐to‐peak amplitude was measured. The *H*_max_ value was expressed relative to the *M*_max_ value (*H*_max_/*M*_max_ (%)) (Chen and Zhou, 2011) to account for variation between participants due to anatomical, conduction, and electrode placement differences (Brinkworth et al., 2007). The stimulation intensity at which the *H*_max_ and *M*_max_ occurred was also recorded and used to describe the level of stimulation required to evoke *H*_max_ relative to the level of stimulation required to evoke *M*_max_ (described as the stimulation intensity ratio). Scores greater than three standard deviation units away from the mean of residuals were considered outliers and removed from the data analysis.

To test the primary hypothesis, paired *t‐*tests were used to compare sitting and standing for the *H*_max_/*M*_max_ and the *H*_max_/*M*_max_ stimulation intensity ratio. However, a noticeable directional trend was noted in the data, so changes in *H*_max_/*M*_max_ (%) were further examined for directionality of change. Eleven individuals displayed an increase in relative *H*_max_ amplitude (termed facilitation) and seven others displayed no change in the relative *H*_max_ amplitude (termed no change). To examine this bidirectional modulation further, a 2 × 2 condition (sitting, standing) × group (facilitation, no change) factorial ANOVA for *H*_max_ and *H*_max_ stimulation intensity (both expressed relative to the corresponding *M*_max_ value) with a Bonferroni adjusted *α* = 0.0125 (.05/4) was completed. This analysis revealed differences between groups for the sitting condition, so a one‐factor analysis of covariance (ANCOVA) was conducted using the sitting condition as the covariate to compare differences in standing. Data were then split by group (facilitation, no change) and one‐factor (condition: sitting × standing) repeated measures ANOVA tests with a Bonferroni corrected *α* = 0.0125 (.05/4) were run to assess within‐group reflex modulation. Finally, separate 2 × 2 (condition × group) factorial ANOVA tests were used to assess the muscle activity of the SOL and TA prior to stimulation.

Bivariate linear regression analyses between sitting and standing conditions were conducted for the facilitation and no change groups separately (Dragert and Zehr, 2013). Analyses were performed on scatter plot recruitment curves using H‐reflex amplitude data from the ascending limb of the recruitment curve, at ≤50% of the stimulation intensity at *M*_max_, as the descending part of the curve is less sensitive to modulation because of antidromic collision (Aagaard et al., 2002; Dragert and Zehr, 2011). This scatterplot recruitment curve approach has been used in previous literature as a method to detect changes in the H‐reflex as the recruitment curve progresses with gradual increases in stimulation intensity to a maximal response (Dragert and Zehr, 2011). Data are presented as Means ± SD, and effect sizes are reported as partial eta squared ($\eta_{p}^{2}$). Data analysis was completed using IBM SPSS version 24.

## Results

As previously mentioned, TA H‐reflexes were undetectable without a background contraction. With a 10% MVC background contraction, H‐reflexes were detected across 18 participants. All data reported below are from responses elicited during a 10% background contraction. To ensure stability of stimulation conditions between sitting and standing for each group, a ratio of the *M*_wave_ associated with the *H*_max_ to the *M*_max_ (*M*_wave_/*M*_max_) was calculated and compared between conditions. A condition (sitting, standing) × group (facilitation, no change) ANOVA did not reveal any differences for the *M*_wave_/*M*_max_ ratio, *F*(1,16) = 1.369, *P* = 0.259, $\eta_{p}^{2}$ = 0.079. Furthermore, data were split by group and separate paired sample *t*‐tests were run comparing sitting and standing. There were no differences between sitting (Facilitation: 32.8 ± 35.1%; No Change: 35.7 ± 36.3%) or standing (Facilitation: 34.9 ± 23.8%; No Change: 20.8 ± 24.4%) for either group (Facilitation: *t*(10) = −0.253, *P* = 0.805; No change: *t*(6) = 1.155, *P* = 0.292).

### Interactions between facilitation and no change

Participants were grouped based on facilitation (*n* = 11) or no change (*n* = 7). A significant condition × group interaction was revealed for modulation of *H*_max_/*M*_max_ *F*(1,16) = 12.751, *P* = 0.003, $\eta_{p}^{2}$ = 0.444, but not for the *H*_max_/*M*_max_ stimulation intensity, *F*(1,16) = 1.083, *P* = 0.314, $\eta_{p}^{2}$ = 0.063. Thus, the amplitude of the reflex relative to *M*_max_ but not the requisite stimulation intensity to evoke *H*_max_ was modulated differently between groups with a change in posture.

There was no significant condition × group interaction for the muscle activity prior to stimulation in the SOL, *F*(1,16) = 0.903, *P* = 0.356, $\eta_{p}^{2}$ = 0.053, or TA muscle, *F*(1,16) = 0.725, *P* = 0.407, $\eta_{p}^{2}$ = 0.043, indicating consistent background EMG activity.

Data were further converted to percent change [((standing‐sitting)/sitting) × 100%] between conditions to simplify the interpretation of the interactions described above. Separate univariate ANOVA tests found a significant difference for the *H*_max_/*M*_max_ percent change from sitting to standing between facilitation (72.6 ± 53.1%) and no change (−23.3 ± 21.2%), *F*(1,16) = 20.405, *P* < 0.001, $\eta_{p}^{2}$ = 0.561. After removing an additional outlier detected in the SOL data of the no change group, separate univariate ANOVA tests failed to detect significant differences between sitting and standing for the percent change level of background SOL activation between facilitation (30.2 ± 84.0%) and no change (17.4 ± 64.0%), *F*(1,15) = 0.114, *P* = 0.740, $\eta_{p}^{2}$ = 0.008, or percent change TA activation between facilitation (14.7 ± 36.2%) and no change (9.9 ± 41.9%), *F*(1,16) = 0.067, *P* = 0.799, $\eta_{p}^{2}$ = 0.004.

### Amplitude modulation (H_max_/M_max_ (%))

**Figure 1:**
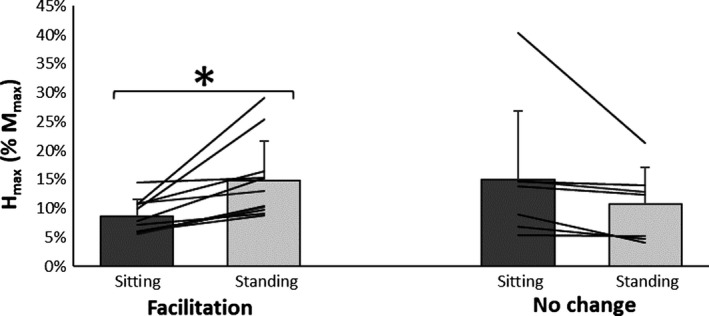
Data represent participants split into groups based on *H*_max_/*M*_max_ ratio modulation response (*n* = 11 facilitation; *n* = 7 no change), there was a significant interaction effect between groups (*P* = 0.003, $\eta_{p}^{2}$ = 0.444). Additionally, there was a significant change in the *H*_max_/*M*_max_ ratio for the facilitation group only (* = 8.7 ± 2.9%, *P* = 0.005, $\eta_{p}^{2}$ 
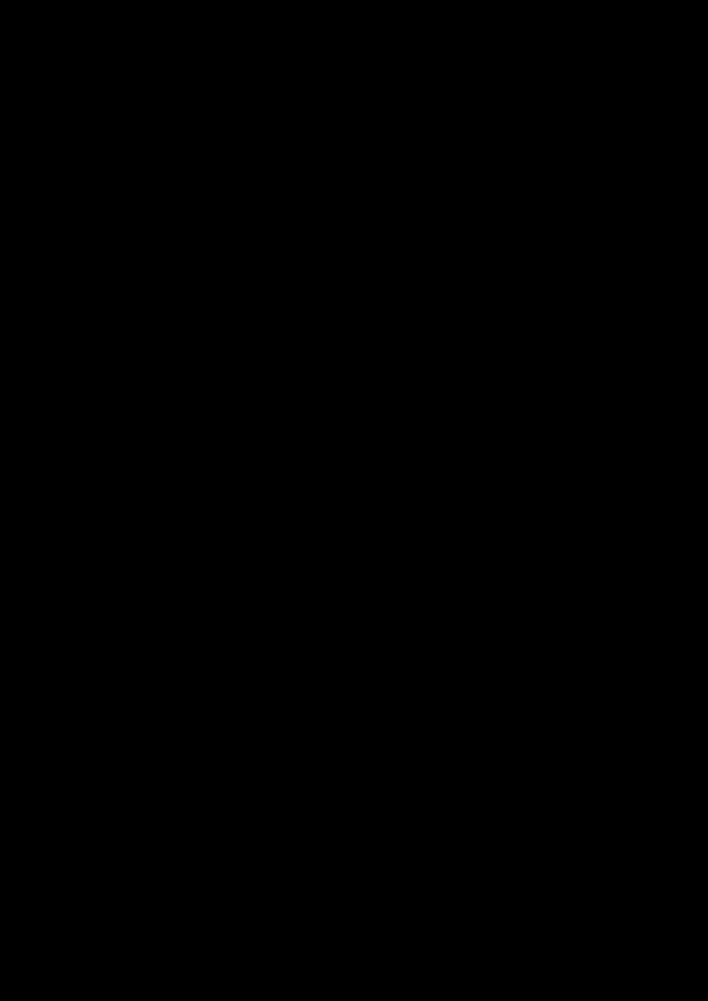
= 0.564).

These data were split by group and separate univariate ANOVA tests were run to compare the change from sitting to standing within each group. A significant effect of condition was found for changes in *H*_max_/*M*_max_ amplitude for the facilitation group only (Fig. 1). The facilitation group demonstrated a significant increase from sitting (8.7 ± .2.9%) to standing (14.8 ± 6.7%), *F*(1,10) = 12.922, *P* = 0.005, $\eta_{p}^{2}$ = 0.564, while there were no significant changes for the no change group between sitting (15 ± 11.8%) and standing (10.7 ± 6.3%), *F*(1,6) = 2.941, *P* = 0.137, $\eta_{p}^{2}$ = 0.329. Additionally, there were no differences between groups for the *H*_max_/*M*_max_ while sitting, *F*(1,16) = 2.978, *P* = 0.104.

### Recruitment curves

**Figure 2:**
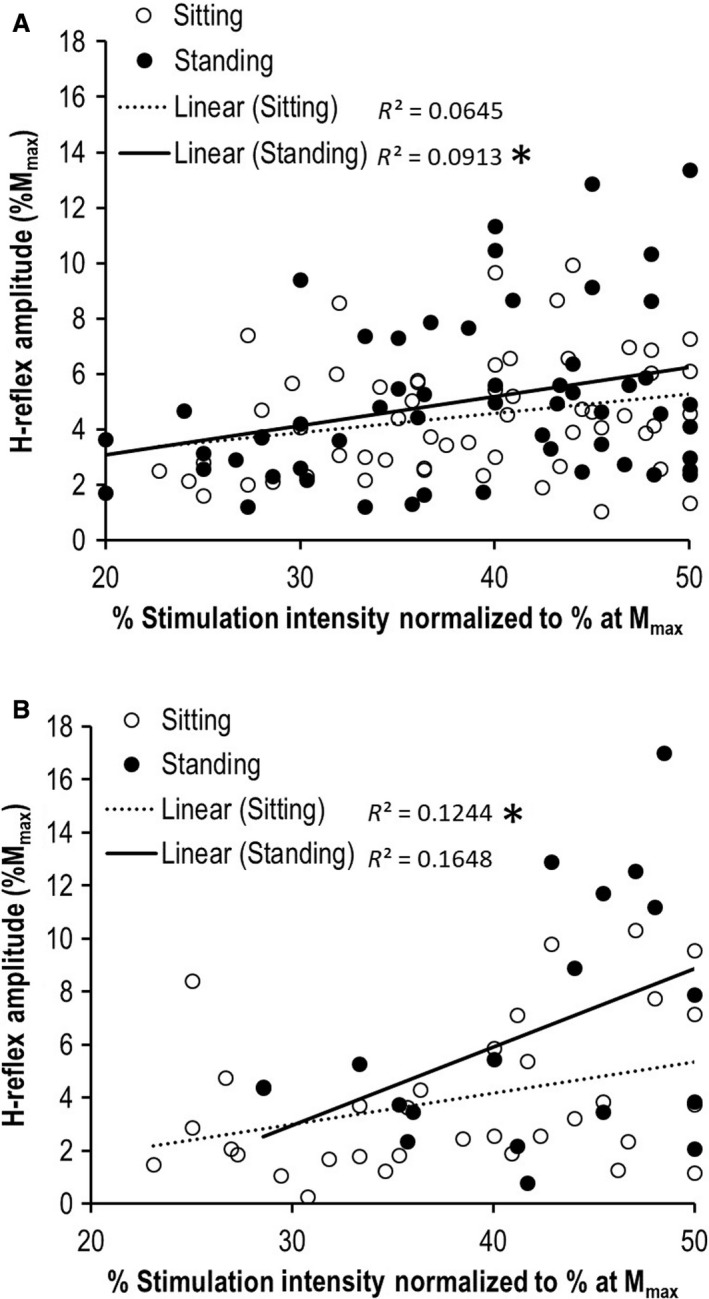
Linear regression analysis on ascending limb of the recruitment curves between conditions (A) Facilitation and (B) No change. Scatter plots were constructed using H‐wave data at ≤50% of the stimulation intensity at *M*_max_ to assess the change in the slope of the recruitment curve between conditions. *P* < 0.05.

A linear regression analysis on the ascending limb of the recruitment curve for the facilitation group (data points; sitting = 57, standing = 55) revealed a nonsignificant regression in the sitting condition, *F*(1,55) = 3.781, *P* = 0.057, *R* ^2^ = 0.064 and a significant regression for the standing condition, *F*(1,53) = 5.325, *P* = 0.025, *R* ^2^ = 0.091 (Fig. 2A). A linear regression analysis on the ascending limb of the recruitment curve for the no change group (data points; sitting = 34, standing = 18) revealed a significant regression for the sitting condition, *F*(1,32) = 4.548, *P* = 0.041, *R* ^2^ = 0.124, while for standing the regression was nonsignificant, *F*(1,16) = 3.156, *P* = 0.095, *R* ^2^ = 0.165 (Fig. 2B).

### Muscle activation prior to stimulation

For the facilitation group, there were no significant differences in background muscle activity prior to stimulation between sitting and standing in the SOL (5 ± 6 vs. 3 ± 4 *μ*V, respectively), *F*(1,9) = 0.555, *P* = 0.475, $\eta_{p}^{2}$ = 0.058, or the TA (8 ± 8 vs. 7 ± 6 *μ*V, respectively), *F*(1,10) = 0.197, *P* = 0.667, $\eta_{p}^{2}$ = 0.019.

Likewise, for the no change group, there were no significant differences in the muscle activity prior to stimulation between sitting and standing in the SOL (8 ± 1.5 vs. 3 ± 4 *μ*V, respectively), *F*(1,6) = 0.810, *P* = 0.403, $\eta_{p}^{2}$ = 0.119, or the TA (1.6 ± 0.1.4 vs. 1.8 ± 1.8 *μ*V, respectively), *F*(1,6) = 0.457, *P* = 0.524, $\eta_{p}^{2}$ $\eta_{p}^{2}$= 0.071.

**Figure 3:**
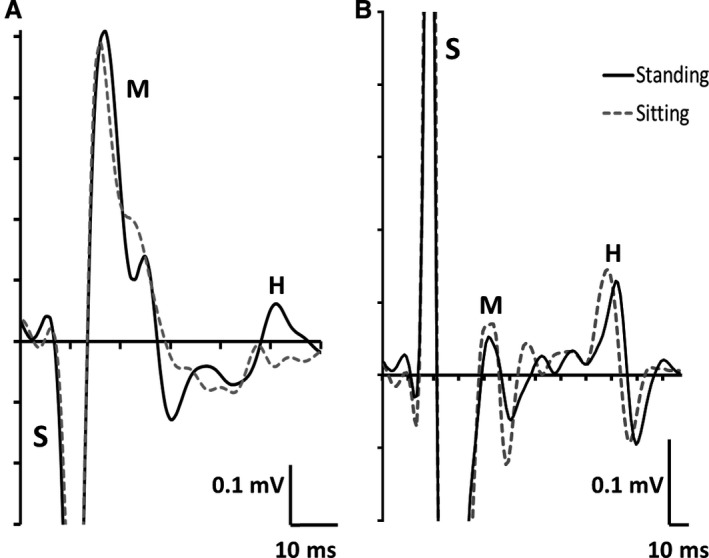
Representative data from each group. (A) Participant from H‐reflex facilitation group, (B) Participant from H‐reflex no change group, demonstrating a change in H‐reflex amplitude between conditions (i.e., sitting, standing) at a matched M‐wave amplitude. S = Stimulation artifact, M = M‐wave, H = H‐reflex.

A representative tracing of the change in TA H‐reflex for each group is displayed in Figure 3.

## Discussion

This experimental work examined changes in relative maximal H‐reflex amplitude and stimulation intensity of the TA when changing from sitting to standing in healthy young adults. A trend emerged where TA H‐reflexes did not change (*n* = 7) or were facilitated (*n* = 11) as posture changed. Bidirectional H‐reflex modulation has been reported during rhythmic arm cycling in neurologically intact participants in the TA (Dragert and Zehr, 2009), in the SOL after stroke (Mezzarane et al., 2014), and in the SOL after either a balance or ballistic strength training intervention when assessed during a functional task (Schubert et al., 2008); however, these studies all showed a split between facilitation and suppression, rather than facilitation and no change, as we see here. The current data add to the literature demonstrating modulation of the TA with different experimental paradigms in various populations. The origin of the modulatory response in TA remains unclear. Different levels of background Ia presynaptic inhibition could influence the magnitude of modulation in either direction (Pierrot‐Deseilligny, 1997). However, since we were unable to elicit H‐reflexes without a background contraction, postsynaptic input may have also been a factor (Pierrot‐Deseilligny and Mazevet, 2000). We attempted to control for this modulation through the standardization of each participant performing a background contraction at 10% of their maximum. Careful future work involving conditioned H‐reflex protocols is needed in order to elucidate such “set points” of background Ia presynaptic inhibition.

Prior work has shown modulation (facilitation or suppression) of reflexes in other muscles of the lower leg with changes in posture, which indicates an important role in maintaining balance and stability (Chen and Zhou, 2011; Kim et al., 2012, 2013). A possible explanation for the findings of the current study is that the direction of modulation reflects one’s proficiency (Schubert et al., 2008) and perhaps strategy for maintaining balance (i.e. spinal reflex versus supraspinal reliance) with increased postural challenge (Taube et al., 2008). Increasing supraspinal activation and suppressing spinal reflex reliance, may be a learned behavior and functional improvement (Huang et al., 2009) resulting in improved balance and stability with increased postural challenge (Taube et al., 2008). For example, prior studies have shown that people who place regular high demand on their balance systems, such as ballet dancers, show increased inhibition of spinal reflexes when compared to other athletes (Nielsen et al., 1993). This difference is possibly due to the demands of ballet training, such as maintaining challenging postures, which lead to specific neural changes directly related to balance control (Ryder et al., 2010).

The H‐reflex is known to be influenced by inputs on presynaptic interneurons from the antagonist muscle, joints, tendons, cutaneous afferents, and supraspinal cortical tracts communicating with the α‐MN (Zehr, 2002; Knikou, 2008). In the current study, while we standardized joint angle, muscle force, and positioning for all participants, joint angle and joint angle changes were not measured during the experiment for each task. While the presynaptic inputs from these sensory sources can influence the H‐reflex, we suggest these factors are important, but unlikely to have caused the modulation we found. Differences in agonist (TA) or antagonist (SOL) background muscle activity were also unlikely to have contributed to the findings, since the level of muscle activity was not different between groups (i.e. facilitation and no change) or conditions (i.e. sitting and standing). This suggests the change due to standing was related to neural modulation and not simply changes in muscle properties aligned with postural requirements. However, we cannot rule out the contribution of disynaptic inhibitory pathways as part of the neural modulation, in addition to changes in presynaptic inhibition, since we did not use techniques to condition the H‐reflex.

Another interesting finding in the current study was a difference in the recruitment curve analyses from sitting to standing, for each group (Fig. 2). Increased stimulation intensity to evoke H reflexes suggests a more active spinal reflex system (Simonsen et al., 2013) and an increased depolarization threshold for maximal motor unit recruitment (Burke et al., 2001). With that, higher stimulation intensity for the *H*_max_ would be required to influence an already active spinal neuronal network during standing. The analyses demonstrated that the slope of the recruitment curve was significant only during the standing condition for the facilitation group (Fig. 2A) and only during the sitting condition in the no change group (Fig. 2B). This suggests the α‐MN depolarization threshold in those conditions was decreased, resulting in a more excitable nervous system, exhibited by a greater H‐reflex amplitude at lower stimulation intensities compared to the other conditions. These results further support the findings of the current study, whereby a linear increase in the ascending limb of the recruitment curve was evident only in the conditions that evoked the highest H_max_ for the respective groups (facilitation = standing; no change = sitting).

### Limitations

Limitations of the present study include minimal demographic information (i.e. prior physical activity levels), lack of continuous ankle angle measurements during testing, and lack of a balance assessment, which makes conclusions on the functional interpretation of these findings difficult. In the standing condition, it might have been useful to know the direction of sway so as to coordinate the timing of the stimulation. Since the TA is an ankle dorsiflexor, reflexes may be modulated from sitting, if the TA was either activated eccentrically, slowing backwards sway, or concentrically initiating forward sway during standing. Due to the exploratory nature of this pilot study, no conclusions can be drawn in regard to the mechanisms responsible for the modulatory responses, warranting further exploration under similar experimental conditions. Of note, under the current experimental conditions, we were unable to reliably detect TA H‐reflexes without a background contraction, and even with background contraction, we obtained reflex data from only 18 of 29 young volunteers.

## Conclusions

In this simple experimental study, we found modulation of the H‐reflex in the TA where some participants showed a facilitation, and others showed no change of the H‐reflex when changing from sitting to standing. The factors contributing to the different modulatory responses in TA during postural change remain unclear. Future research would benefit from the addition of EMG activity in the contralateral limb for the homonymous (TA) and heteronymous (SOL) muscles in addition to recording muscles proximal to the TA in the ipsilateral limb as these data may provide insight into the cause of the modulation response. Additionally, future research should consider implementing a valid balance assessment task as part of the data collection to directly measure balance control and/or consider the direction of sway when measuring the H‐reflex. Furthermore, investigating the reflex contributions in varying postural conditions (e.g., lying down, sitting, standing, walking), muscles, and tasks, will help further understanding of the mechanisms and function of reflex modulation in the lower limb. Currently, most of the literature investigating H‐reflex modulation has targeted the SOL. There is a lack of understanding about how the spinal and supraspinal presynaptic inputs onto the α‐MN are modulated with different postural conditions and tasks in the TA. The current study provides preliminary evidence of the possible bidirectional influence of standing posture on Ia presynaptic inhibition and serves as a base from which future research can expand.

## Conflicts of Interest

None.
